# Factors Associated with Anxiety and Depression among African American and White Women

**DOI:** 10.5402/2012/432321

**Published:** 2012-01-03

**Authors:** Kalycia Trishana Watson, Nehezi M. Roberts, Milda R. Saunders

**Affiliations:** ^1^Department of Urban Planning and Policy, University of Illinois at Chicago, 412 S. Peoria, Room 215, MC 348, Chicago, IL 60607, USA; ^2^University of Chicago School of Social Service Administration, Chicago, IL 60637, USA; ^3^University of Chicago Medical Center, 5841 South Maryland Avenue, MC 5000, Chicago, IL 60637, USA; ^4^Section of Hospital Medicine and MacLean Center for Medical Ethics, University of Chicago Medical Center, 5841 South Maryland Avenue, MC 5000, Chicago, IL 60637, USA

## Abstract

*Background.* We examined factors associated with depression and anxiety in a cohort of low-income Baltimore women. *Methods.* We used Pathways to Adulthood data, a cohort of adults aged 27 to 33 who were born in Baltimore between 1960 and 1965. Our outcomes were a score of >4 on the General Health Questionnaire (GHQ-28) across the depression or anxiety domains. Linear regression clustered on census tract was used for multivariate analysis. *Results.* In multivariable analyses, unmarried women, White women, those with lower self-rated health, and younger mothers had higher depression scores. Only lower self-rated health and White race were associated with a higher anxiety score. Neither neighborhood poverty nor racial composition was a predictor for anxiety or depression; however, the significant risk factors cluster in disadvantaged neighborhoods. *Conclusion.* Our work highlights the importance of universal screening for depression or anxiety with more in-depth surveillance based on risk factors rather than on race.

## 1. Introduction

Depression and anxiety are common mental health disorders; however, the risks for depression and anxiety are not distributed equally. Women are twice as likely as men to experience depression and generalized anxiety disorder due to a variety of biological and social factors [1, 2]. Women's hormonal fluctuations associated with menstruation, pregnancy, and childbirth may increase their vulnerability to mental distress. Social factors such as poverty, race, and neighborhood also increase women's risk for depression and anxiety.

Poverty is considered one of the most consistent predictors of mental distress among women. Low-income women's risk of depression is almost double that of their nonpoor counterparts [2, 3]. Poverty contributes to daily worries about basic needs and limits the ability to engage in recreational activities. Poor women are more likely to experience financial troubles, relationship problems, poor health, and unemployment which can contribute to the onset of depression [3–5]. Poverty may combine with a lack of social support to make low-income women vulnerable to anxiety and depressive disorders.

For women, racial differences in risk for depression and anxiety vary widely. In some studies, African American women have higher rates of depression and anxiety compared to White women [6], while other data demonstrate that African American women have lower rates [2, 7–11]. Lower rates of anxiety and depression among African American women may be attributed to psychosocial resources, emotional resilience, social support, and ethnic identity [6]. Higher rates in African American women are generally attributed to greater levels of poverty, poor health, and stigma about mental health care [12–14]. Anxiety disorders among African American women are particularly understudied, which contributes to uncertainty about its risk factors and rates in this population [15]. Neighborhood poverty is also an understudied risk factor for depression and anxiety in women [16]. Neighborhood disadvantage can obstruct the creation of bonds among residents, reducing social support and social control that serve as a protective factor against psychological and economic stressors [17]. By increasing daily hassles and reducing protective factors, poor neighborhoods increase an individuals' vulnerability to depression when negative events occur [18]. Women who live in poor neighborhoods have fewer opportunities for employment, education, recreational activities, and positive social interactions which can cause or exacerbate depression or anxiety [19]. African American women are more likely to live in poor neighborhoods than their White counterparts [20].

To date, few studies have been able to compare rates of depression and anxiety in African American and White women while examining both individual and neighborhood characteristics. We used a unique cohort of African American and White women from the Pathways to Adulthood, a follow-up of a longitudinal cohort of inner city women and their children in Baltimore, Md, USA. Our aims were to (1) examine what individual and neighborhood socioeconomic factors are associated with depression and anxiety and (2) determine whether these relationships varied between African American and White women.

## 2. Methods

### 2.1. Study Population

The Johns Hopkins Perinatal Collaborative Study in conjunction with the Pathways to Adulthood follow-up was a retrospective study of three generations of families initially living in the inner city Baltimore. The Perinatal Collaborative Study enrolled 2307 inner-city women (referred to throughout as first-generation mothers (G-1s)) who were selected at random at the time of their first prenatal visit to a public obstetric clinic at Johns Hopkins Hospital between 1959 and 1965 [21]. The women lived within a 10-block radius of the hospital. The study later collected data on the 2694 children of these first-generation mothers who were born between 1960 and 1965 (children referred to as second generation (G-2s) throughout). These children were initially studied prospectively with data gathered between birth and 8 years of age regarding their neurologic and cognitive development, health, behavior, and family, and neighborhood socioeconomic characteristics [22].

From 1992 to 1994, the Pathways to Adulthood Study collected additional information from the 1758 G-2s (then aged 27 to 33) about their lives from age 9 to present. Follow-up data included information on education, employment, family composition, health, health care usage, and income [21]. In addition to these data that were collected directly from the G-2 individuals, data regarding the neighborhood characteristics of each G-2 at birth, at 11-12 years of age, at 16-17 years of age, and at age of follow-up (ages 27–33) were obtained through census data. For a full description of sample population and methods, see [22]. Our final sample (*n* = 989 G2 females) included the 75% of female respondents who provided information (in-person or via telephone) for the Pathways to Adulthood Study and had information on the depression or anxiety measures.

### 2.2. Outcome Variables

The General Health Questionnaire (GHQ-28) is a self-administered screening questionnaire aimed to detect probable psychiatric disorders in primary care settings [23]. The threshold for a “case” (individuals who “probably need further evaluation”) is 4-5 depending on the population [24]. For greater specificity, we defined a case as a GHQ score greater than or equal to 5. The GHQ-28 has four subscales: somatic, anxiety, social dysfunction, and depression and that each contains seven questions [25]. The subscales provide individual diagnostic or profile information; however, they have no diagnostic thresholds. For our study, a GHQ depression subscale score greater than or equal to 4 was defined as “severe depression” and a GHQ anxiety subscale score greater than or equal to 4 was defined as “anxiety.”

### 2.3. Covariates

#### 2.3.1. Demographic and Health-Related Characteristics

We examined individual characteristics (including demographic age, race, number of children, and marital status) and health-related characteristics (current drinking status and self-reported health rating).

#### 2.3.2. Socioeconomic Status

We used assets, education, and income to quantify socioeconomic status. We treated years of education as a continuous variable. Although we had two measures for income, self-reported total household income and personal income, the high rate of missing values in both measures precluded their use in the analysis. We treated assets as a continuous variable ranging from 0 to 6, with one point given for a “yes” response when asked about each of six assets (current personal checking account; current IRA or pension, own house or condo, car, truck, or motorcycle ownership, credit or charge account, and current savings account). After factor analysis showed home ownership to be the most robust measure, we included homeownership in our model.

#### 2.3.3. Neighborhood Characteristics

Individuals' neighborhood characteristics were obtained from the 1990 census. Each respondent's address at the time of the interview was linked to data from the appropriate census tract. We reported neighborhood household income as a continuous variable. We based neighborhood racial composition on percentage of African American residents. We also categorized neighborhoods based on percent of respondents below the federal poverty level.

### 2.4. Analyses

We used cross-tabulations to compare all categorical variables by race, neighborhood, and weight status. We used chi-square statistics as the corresponding measure of heterogeneity. For continuous variables, we determined summary measures (mean and standard error) for each sub-group. We used analysis of variance (ANOVA) to compare mean values across subgroups. First, we examined the bivariate association between race and our covariates of interest. Next, we used linear regression to examine the multivariate associations between our outcomes—depression and anxiety subscale scores and the covariates of interest. During this process, we included those in which we had substantive a priori interest based on prior literature and included those that were of at least borderline statistical significance during forward stepwise selection (*P* < 0.10). For multivariate analysis, we used linear regression clustered on census tract while controlling for marital status, race, age at first child, number of children, years of education, homeownership, neighborhood poverty, and percent African American in the neighborhood. The point estimate is the same as in nonclustered linear regression; only the standard error is corrected. All analyses were done using Intercooled Stata (version 11.0; Stata Corporation, College Station, Tex, USA).

## 3. Results

### 3.1. Baseline Characteristics by Race

A summary of baseline sociodemographic, health, and neighborhood characteristics of the cohort is presented in Table 1. The sample was 18% White and 82% African American. They differed significantly on measures of psychological distress. White women scored significantly higher than African American women on total GHQ score (4.42 versus 3.12), mean depression score (0.57 versus 0.25), and mean anxiety score (1.80  versus 1.22), all *P* < 0.05.

Although household income was not significantly different between White and African American women ($35,638 versus $31,807, *P* = 0.18), they differed on many other socioeconomic indicators. Compared to their White counterparts, African American women had more years of education (12.87 versus 10.89 for Whites), were more likely to have completed college (14% versus 3%), and had higher personal income (*P* < 0.05). White women had a higher mean number of assets than their African American counterparts (3.17 versus 2.48, *P* < 0.05), and African American and White women did not differ on the number of children or age of first child (*P* > 0.05). However, White women were significantly more likely to be married (59% versus 27%, *P* < 0.05), which may, in part, account for the differences in both assets and household income.

African American and White women also substantially differed by current neighborhood characteristics (Table 1). White women lived in neighborhoods with an average household income that was significantly higher than African Americans ($37,578 versus $28,655). White women were less likely to live in poor neighborhoods (25% versus 58%) or to report a concern with crime (25% versus 36%), all *P* < 0.05. The neighborhoods in which the African American study participants lived had a significantly higher proportion of African Americans than their white counterparts. On average, White women lived in neighborhoods that were 8% African American, while African women lived in neighborhoods that were predominantly African American (72%).

### 3.2. Comparison by GHQ Score

Approximately 30% of the sample had a GHQ score greater than or equal to 5 (Table 2). Women who met the GHQ case definition had a lower socioeconomic status. They had fewer years of education (12 versus 12.7), fewer assets (2.2 versus 2.8), lower family income ($29,790 versus $33,505), and personal income ($12,520 versus $16,487), all *P* values <0.05. They also fare worse on many health measures. Women with a greater GHQ score had a higher BMI (27.3 versus 26.1), were more likely to be smokers (52% versus 42%), and were less likely to report good or excellent health (32.1 versus 65.2), all *P* values <0.05. They did not differ significantly on neighborhood characteristics. Overall, over half the population in both groups lived in poor neighborhoods that were predominately African American, and a significant proportion had concerns about crime within their neighborhoods. In multivariate logistic models, only White race and poor self-rated health were associated with a higher GHQ score. Women with good or excellent health were 75% less likely to have a GHQ score greater than 4 (OR 0.25, 95% CI 0.17–0.36). African American women were almost 50% less likely than their White counterparts to have a positive GHQ score (OR 0.56, 95% CI 0.32–0.99).

### 3.3. Comparison by Depression Status

Women with higher depression scale ratings (≥4) differed significantly from their less depressed counterparts (Table 3). More depressed women had fewer years of education (10.9 versus 12.6) and were less likely to have a college degree (0% versus 12%), all *P* < 0.05. Depressed women had lower assets (0.8 versus 2.7), lower household income ($21,300 versus $32, 633), and lower personal income ($8,591 versus $15,482), all *P* < 0.05. Depressed women were less likely to be married (8% versus 34%) and had a lower age at first child's birth (18.7 versus 21.2), all *P* < 0.05. Women with greater depression scores also had worse overall health. They had a higher BMI (29 versus 26), were more likely to be drinkers (64% versus 44%), and less likely to report good health (24% versus 57%), all *P* < 0.05. Depressed women lived in neighborhoods with lower median household incomes ($24,594 versus $30,045, *P* < 0.05). However, they did not differ significantly from their counterparts on other neighborhood characteristics such as percent African American, neighborhood poverty, and neighborhood crime.

### 3.4. Comparison by Anxiety Status

Women with higher anxiety ratings (≥4) differed significantly from their less anxious counterparts (Table 4). Women with a high anxiety score were less likely to be African American (76% versus 83.6%), had fewer years of education (11.9 versus 12.6), and had more children (1.72 versus 1.47), all *P* < 0.05. In addition, more anxious women had fewer assets (1.9 versus 2.76), lower family income ($26,325 versus 33,654), and lower personal income ($12,336 versus 15,917), all *P* < 0.05. More anxious women were more likely to be smokers (53% versus 43%) and less likely to report very good or excellent health (34% versus 60%), all *P* < 0.05. Neighborhood characteristics did not differ significantly between women based on anxiety status.

### 3.5. Multivariable Models

Table 5 shows the factors associated with score in GHQ depression domain. Decreased self-rated health, lower age at first child, being unmarried, and White race were significantly associated with a higher depression score. In multivariable analysis, women who were African American (*B* = −0.44), were married (*B* = −0.21), had older age at first child (*B* = −0.02), or reported good or excellent health had lower GHQ depression scores, all *P* < 0.05. A 30-year-old White unmarried woman who had her first child at age 16 would have an average depression score of 0.95 (95% CI 0.71–1.20), while an African American woman under the same circumstances would have an average score of 0.48 (95% CI 0.33–0.62). A 30-year-old woman who was married and had her first child at 21 would have a depression score of 0.62 (95% CI 0.42–0.82) if she were White and 0.13 (95% CI 0.02–0.29) if she were African American.

Table 5 shows factors associated with score on the GHQ anxiety domain. African American women (*B* = −0.77), those with older age at first child (*B* = −0.06), and those who reported good or excellent health (*B* = −0.99) had lower anxiety scores, all *P* < 0.05. The average White woman in our sample had an anxiety score of 1.72 (95% CI 1.29–2.14) if she reported good or excellent self-rated health and 2.7 (95% CI 2.27–3.14) if she did not. The average African American woman in our sample had an anxiety score of 0.81 (95% CI 0.49–1.13) if she reported good or excellent health and 1.8 (95% CI 1.45–2.15) if she did not.

## 4. Discussion

In our study African American women had significantly lower mean depression scores compared to White women. Our work is consistent with a number of studies that have shown that African Americans have lower rates of depression than their White counterparts [2, 7–11]. Although African American women live with greater stress than White women, their coping strategies may make them less vulnerable to the impact of chronic strains [25, 25].

Additionally, our study indicated African American women had significantly lower anxiety scores than White women. Very little research has focused on the rates of generalized anxiety disorder among African American women and White women. A large portion of anxiety research focuses on specific population subgroups (e.g., postmenopausal women, mothers of preterm infants) and subcategories of anxiety (e.g., women with HIV, social phobia, etc.). Consensus has not yet been reached on African American prevalence and presentation of anxiety disorders [15, 26].

Our study is one of the few to report a decreased risk of both depression and anxiety for African American women compared to White women. For African American and White women in similar circumstances, African American women consistently had lower rates of both depression and anxiety. However due to historic and ongoing socioeconomic disparities, African American women experience significantly more challenging life experiences than their White counterparts (i.e., discrimination, neighborhood poverty, and low income) [27, 28].

Unmarried women and women who had children at a younger age had higher depression scores. This is consistent with the literature that shows that adolescent mothers have an increased rate of depression and anxiety due to stressful life events [29, 30]. Prior work also shows that marriage provides economic, social, and psychological support, which contributes to a person's well-being. These social supports and structured routine can encourage healthy behaviors and discourage harmful ones [8, 31, 32].

Health status was also associated with depression and anxiety scores. Women with worse self-reported health had higher scores of depression and anxiety. Poor health can increase daily hassles and reduce pleasure, increasing one's risk for mental distress [5, 13]. Conversely, depression and anxiety can increase one's perception of poor health and can worsen outcomes for preexisting conditions [33]. Determining causality is beyond the scope of this study. However, simply knowing what factors are associated with greater psychological distress can still lead to better efforts to screen for depression and to target mental health services.

### 4.1. Strengths and Limitations

One limitation was our use of the General Health Questionaire-28 for diagnosis. The GHQ-28 was developed to determine people in a general population who need further evaluation for mental health disorders. The threshold for a case, those who “probably need further evaluation,” is 4-5, depending on the population [24]. We used similar cut-offs for the depression and anxiety scale of greater than or equal to 4 to determine diagnosis. There are no specific cut-offs for the subscale domains. Despite this, higher subscale scores reflect increased depressive or anxiety symptoms.

Another important limitation is its generalizability. The Pathways to Adulthood data had a limited geographic focus, children born in the Johns Hopkins Hospital catchment area. This group had a higher proportion of African Americans and higher poverty rates than the USA as a whole. Our findings suggest racial differences in anxiety and depression may be due, in part, to differences in socioeconomic status or neighborhood environment.

One strength of this study is the use of census data for aggregate neighborhood measures. Using a different data source to determine individual and group measures avoids one source of error because the study population may not be a representative sample of the population [34]. An additional limitation, also faced by other researchers studying neighborhood, was characterizing the neighborhood environment solely through some proxy measures based on census data. Despite the wealth of census information, other variables that may be more directly associated with depression or anxiety were absent [35]. Asset mapping may allow a fuller characterization of neighborhood and a deeper understanding of what factors confer health risks.

### 4.2. Conclusion

In this cross-sectional study of African American and White women with similar rates of poverty, we sought to quantify the association between measures of anxiety and depression and individual and neighborhood socioeconomic status and to determine whether these relationships varied by race. At similar levels of disadvantage, White women had higher scores for depression and anxiety. We found an increased risk for depression in unmarried women and those with younger motherhood. Women with lower reported self-rated health had higher scores for both depression and anxiety. Neither neighborhood poverty nor racial composition was in and of itself a predictor of greater risk for anxiety or depression; however, the significant risk factors cluster in disadvantaged neighborhoods. Our work highlights the importance of universal screening for depression or anxiety with more in-depth surveillance based on risk factors rather than on racial classification.

## Figures and Tables

**Table 1:** Comparison of participant characteristics by race.

	White females (*n* = 163)	Black females (*n* = 757)	*P* value
Demographic characteristics			
Age (Standard deviation, SD)	29.9 (0.11)	30.1 (0.05)	0.12
Years of education	10.89 (0.16)	12.87 (0.13)	0.0001
Percentage with college degree	3%	14%	0.0001
Number of assets	3.17 (0.17))	2.48 (0.08)	0.0002
Average family income	$35,638 (2594)	$31,807 (1088)	0.18
Average personal income	$12, 325 (1018)	$16,010 (500)	0.0013
Number of children	1.55(0.09)	1.49 (0.05)	0.54
Percentage married	59%	27%	0.0001
Age at first child's birth	21.19 (0.33)	21.19 (0.16)	0.99
Percent below poverty level	28%	33%	0.216
Health characteristics			
BMI	26.51(0.48)	26.82 (0.22)	0.56
Percent current smoker	52%	42%	0.015
Percent current drinker	85%	84%	0.871
Percent with very good/excellent self-reported health	53%	56%	0.026
Adult neighborhood characteristics			
Median household income	$37,578 (1211)	$28,655 (451)	0.0001
Percent african american	8%	72%	0.0001
Percent poor neighborhood	25%	58%	0.0001
Percent reporting crime concerns	25%	36%	0.010
Psychological characteristics			
Total GHQ-28 score	4.42 (0.38)	3.12 (0.16)	0.002
Mean GHQ depression score	0.57 (0.095)	0.25 (0.03)	0.001
Mean GHQ anxiety score	1.80 (0.17)	1.22 (.068)	0.002

**Table 2:** Comparison of participant characteristics by GHQ case status.

	GH < 5 (*n* = 694)	GHQ ≥ 5 (*n* = 292)	*P* value
Demographic characteristics			
Age (Standard deviation, SD)	30.2	30	0.12
Years of Education	12.7	12	0.003
Percentage with college degree	13.83	8.56	0.02
Number of assets	2.8	2.2	0.0003
Average family income	$33505	$29790	0.09
Average personal income	$16487	$12520	<0.001
Number of children	1.47	1.60	0.18
Percentage married	34.32	29.56	0.16
Age at first child's birth	21.4	20.7	0.04
Percent black	77.40	84.29	0.01
Percent home ownership	26.4	20.6	0.06
Health characteristics			
BMI	26.1	27.3	0.009
Percent current smoker	41.5	52.2	0.003
Percent current drinker	85.5	83.8	0.62
Percent with very good/excellent self-reported health	65.2	32.1	<0.001
Adult neighborhood characteristics			
Median household income	$29829	$30075	0.79
Percent african american	64.9	55.8	0.001
Percent poor neighborhood	54.3	51.8	0.51
Percent reporting crime concerns	33.08	35.45	0.49
Psychological characteristics			
Total GHQ-28 score	1.0	9.4	<0.001
GHQ depression	0.36	3.8	<0.001
GHQ anxiety	.04	1.0	<0.001

**Table 3:** Comparison of participant characteristics by depression status.

	Depression scale < 4 (*n* = 943)	Depression scale ≥ 4 (*n* = 43)	*P* value
Demographic characteristics			
Age (Standard deviation, SD)	30.11 (1.49)	29.78 (1.44)	0.27
Years of education	12.56 (3.52)	10.92 (2.47)	0.02
Percentage with college degree	12.83	0	0.012
Number of assets	2.67 (2.17)	0.76 (1.45)	<0.0001
Average family income	$32,633 (22945)	$21,300 (14614)	0.14
Average personal income	15,482 (12238)	8591 (10719)	0.02
Number of children	1.50 (1.31)	1.72 (1.4)	0.42
Percentage married	33.62	8.00	0.007
Age at first child's birth	21.23 (3.83)	18.70 (2.63)	0.006
Percent black	82.61	74.42	0.169
Health characteristics			
BMI	26.41 (6.01)	29.15 (10.07)	0.03
Percent current smoker	84.28	100	0.061
Percent current drinker	44.00	64.0	0.047
Percent with very good/excellent self-reported health	56.54	24.00	<0.001
Adult neighborhood characteristics			
Median household income	30,045 (12492)	24594 (10335)	0.043
Percent african american	62.32 (37.07)	58.61 (44.12)	0.65
Percent poor neighborhood	53.19	68.18	0.164
Percent reporting crime concerns	33.30	52.17	0.059
Psychological characteristics			
Total GHQ-28 score	3.0 (4.03)	17 (4.43)	<0.001

**Table 4:** Comparison of participant characteristics by anxiety status.

	Anxiety scale < 4 (*n* = 811)	Anxiety scale ≥ 4 (*n* = 175)	*P* value
Demographic characteristics			
Age (Standard deviation, SD)	30.13 (1.50)	29.96 (1.42)	0.18
Years of education	12.6 (3.7)	11.9 (2.2)	0.014
Percentage with college degree	13.19	8.00	0.058
Number of assets	2.76 (2.18)	1.90 (1.99)	<0.0001
Average family income	33,654 (23317)	26325 (19429)	0.0056
Average personal income	15917 (12564)	12336 (10009)	0.0026
Number of children	1.47 (1.32)	1.72 (1.22)	0.028
Percentage married	34.17	26.75	0.071
Age at first child's birth	21.39 (3.86)	20.13 (3.48)	0.0007
Percent black	83.60	76.00	0.017
Health characteristics			
BMI	26.37	27.00	0.25
Percent current smoker	42.9	52.9	0.021
Percent current drinker	85.18	83.96	0.76
Percent with very good/excellent self-reported health	60.03	33.76	<0.001
Adult neighborhood characteristics			
Median household income	30,001 (12751)	29425 (11030)	0.61
Percent african american	63.56 (36.7)	55.85 (39.2)	0.023
Percent poor neighborhood	55.54	53.79	0.955
Percent reporting crime concerns	32.91	38.16	0.210
Psychological characteristics			
Total GHQ-28 score	1.83	11.21	<0.0001

**Table 5:** Multiple linear regression for increasing score in depression and anxiety domain.

	Depression (*β*)/95% CI	Anxiety (*β*)/95% CI
Individual characteristics		
Black race	−0.44 (−0.72, −0.17)*	−0.77 (−1.33, −0.20)*
Age	−0.02 (−0.05, 0.03)	−0.09 (−0.19, 0.02)
Age at first child	−0.02 (−0.05, −0.003)*	−0.06 (−0.11, −0.02)*
Number of children	0.006 (−0.07, 0.08)	−0.08 (−0.24, 0.81)
Married	−0.21 (−0.39, -0.03)*	−0.22 (−0.60, 0.15)
Good or excellent health	−0.41 (−0.55, −0.26)*	−0.99 (−1.30, −0.68)*
Homeowner	−0.61 (−0.26, 0.14)	−0.19 (−0.60, 0.22)
Neighborhood characteristics		
Percent african american	−0.001 (−0.005, 0.002)	−0.003 (−0.01, 0.003)
High poverty neighborhood	0.11 (−0.09, 0.30)	0.08 (−0.31, 0.48)

*P < 0.05.
